# Demand for long acting contraceptive methods and associated factors among family planning service users, Northwest Ethiopia: *a health facility based cross sectional study*

**DOI:** 10.1186/s13104-015-0974-6

**Published:** 2015-02-04

**Authors:** Saleamlak Adbaru Yalew, Berihun Megabiaw Zeleke, Alemayehu Shimeka Teferra

**Affiliations:** Teda Health Science College, Teda Town, P.O. Box 790, Gondar, Amhara Regional State Ethiopia; Department of Epidemiology and Biostatistics, College of Medicine and Health Sciences, University of Gondar, P.O. Box 196, Gondar, Ethiopia

**Keywords:** Demand, Unmet need, Long acting contraceptives, Debre-Tabor town

## Abstract

**Background:**

Demand for long acting contraceptive methods is one of the key factors for total fertility rate and reproductive health issues. Increased demand for these methods can decline fertility rate through spacing and limiting family size in turn improving maternal and family health and socioeconomic development of a country. The aim of this study was to assess demand for long acting contraceptives and associated factors among family planning users in Debre-Tabor Town, Northwest Ethiopia.

**Methods:**

Facility based cross-sectional study was conducted from July to August 2013. Data was collected on 487 current family planning users through face to face interview using structured questionnaire. Study participants were selected by systematic sampling method. Data were entered in to Epi Info and analyzed by using SPSS version 20. Bi-variable and multi-variable regression analyses were done to identify factors associated with demand for long acting contraceptive methods. Odds ratio with 95% CI was used to assess the association between the independent variables and demand for long acting family planning methods.

**Results:**

The study showed that, demand for long acting contraceptives was 17%. Only 9.2% of the women were using long acting contraceptive methods (met need). About 7.8% of women were using short acting methods while they actually want to use long acting methods (unmet need). Demand for LACMs was positively associated 3with being a daily labour (AOR = 3.87, 95% CI = [1.06, 14.20]), being a student (AOR = 2.64, 95% CI = [1.27, 5.47]), no future birth intensions (AOR = 2.17, 95% CI = [1.12, 4.23]), having five or more children (AOR = 1.67, 95% CI = [1.58, 4.83]), deciding together with husbands for using the methods (AOR = 2.73, 95% CI = [1.40, 5.32]) and often having discussion with husband (AOR = 3.89, 95% CI = [1.98, 7.65]). Clients treated poorly by the health care providers during taking the services was negatively associated with demand for LACMs (AOR = 0.42, 95% CI = [0.24, 0.74]).

**Conclusion:**

Demand for long acting family planning methods was observed to be lower as compared to other studies. There were also significant proportion of women having unmet need for long acting methods – women using short acting method while actually wanting long acting methods. Therefore, it is necessary to create and increase awareness and advocacy on demand for long acting contraceptive methods considering women and their husbands. Moreover, emphasis should be given to service provision of the methods.

## Background

The use of contraceptive methods has become an essential factor in the life of most reproductive age group of women, although it varies in different points of their life course. In order to promote women’s reproductive health and prevent the risk of unwanted pregnancies, the use of effective contraceptive methods is paramount important. Demand of long-acting contraceptive methods is one of the key factors to protect women and couples against unwanted pregnancies. However, the proportion of women using these methods was lower than the proportion who desires to use the methods. Increasing demand for these methods will improve utilization of the methods; in turn, it will decline total fertility rate. If demand is not met, on the other hand, the number of unwanted pregnancy and abortion is more likely to increase [1,2].

Sub Saharan Africa, including Ethiopia faces serious population and reproductive health challenges, which is indicated by higher maternal mortality, higher total fertility and population growth rate, and higher unmet need for family planning. In many Sub-Saharan countries, there is a higher proportion of unmet need for family planning specially for long acting methods. This problem is often more common among rural women. Though, there is the intention to increase the utilization of long acting contraceptive methods, a significant proportion of women are using short term family planning methods in the region [2,3].

Every year approximately 350,000 women die while pregnant or giving birth. Of these women, 99% die in developing countries. An estimated 8 million more women suffer serious illnesses and lifelong disability as a result of complication during childbirth. As evidences shown, Ethiopia is one of the country with unacceptably higher level of maternal mortality, currently 676 per 100,000 live births [3,4].

The total population of Ethiopia is estimated to be over 85 million with the total fertility rate of 4.8 children per reproductive age group women; this high fertility rate, makes Ethiopia Africa’s second most populous country next to Nigeria. Family planning service delivery facilities and other supplies have increased in number in the country; however, significant portion of the Ethiopian population is still living in abject poverty of family planning service [4,5].

Creating wider access to family planning especially long acting contraceptive methods which are the relatively effective contraceptives than other methods can substantially reduce unwanted pregnancy and unsafe abortions in turn the higher level of pregnancy related maternal mortality in developing countries. The cultural and religious background of a given community has powerful influence on health seeking behaviour in general and contraceptive use in particular. Studies suggest that there is strong opposition against utilization of long acting contraceptive methods from the religious institutions [1,4].

Population growth is the major global problem. Fertility rate in Ethiopia is one of the highest in the world and the utilization of long acting contraceptive methods was very low. According to EDHS 2011 about 25% of currently married women have an unmet need for family planning 16% for spacing and 9% for limiting. The proportions of women currently using long acting contraceptive methods were significantly lower than the proportion of those using short-acting contraceptive methods [3,4].

Long-acting reversible contraceptives are methods that provide effective contraception for an extended period of time without requiring the user’s action and with low burden to the users after initiation. They include intrauterine devices (IUDs) and sub dermal implants and allow a rapid return to fertility after their removal. They are cost effective, can result in substantial cost savings for governments, and contribute directly to reaching national and international health goals [6-8].

According to the Debre-Tabore town Health Department report of 2012 utilization of long acting contraceptive methods was very small. Among the total family planning service user women, only 2.6% and 4.6% were taking IUCD and implanol, respectively [9]. Studies has been conducted on demand for long acting family planning methods in the general population, however, there was no research on unmet need and demand for long acting contraceptive methods among family planning users. Similarly, there was no study conducted to assess the level of demand for long acting contraceptives methods and associated factors. Therefore, this study aimed to assess the level of demand for long acting reversible contraceptives and determinant factors among family planning users in Debre-Tabore Town, Northwest Ethiopia. The findings from this study would help policy makers and planners and other concerned organizations working in the area of family planning and maternal health.

## Methods

### Study design and setting

Facility based cross-sectional study was conducted to assess the level of demand for long acting contraceptive methods and associated factors among family planning service users in Debre-Tabore town, Northwest Ethiopia. The study was conducted from July to August, 2013. The town has four administrative kebeles (the lowest governmental administrative units) with a total population of 67,485; among these 13,969 were in the reproductive age group. There is one zonal hospital, three health centers, two medium private clinics and one small scale private clinic, all providing family planning service in the town. All family planning service users mothers aged from 15-49 years who are residents of the town were taken as source population [10].

### Sample size and sampling procedure

The sample size was calculated using EPI info version 3.5.3 assuming that estimated proportion of demand for long acting and permanent contraceptive methods from a previous study done in Batu town, Eastern Shoa Zone as 24.4% [11], 95% level of confidence, and 4% of margin of error. The calculated sample size was 443 but by considering a 10% non-response rate, the final required sample size was taken to be 487.

### Sampling procedure

Both public and private health facilities were providing family planning services in the town. Purposively, for this study only government health facilities were considered, i.e. all of these government health facilities; Debre-Tabor hospital, Debre-Tabore, Ginbot 20 and Hidar 11 health center. The calculated sample size was proportionally allocated to each health facility based on the previous consecutive three months average daily client flow of the units which were obtained by referring client registration log books. The average monthly client flow for Debre-Tabor hospital, Debre-Tabore health center, Ginbot 20 health center and Hidar 11 health center were 900, 600, 450 and 450, respectively. The average monthly client flow was 2400 women were had been taking contraceptive methods in all the four selected health facilities during data collection period. The study participants were selected by using systematic random sampling method from family planning service users who visited the health institutions during the data collection period. The first client in each health facility was selected by lottery method.

### Operational definitions

#### Long acting contraceptive methods

In this study only the reversible long acting contraceptives methods such as Intra-Uterine contraceptive Device (IUCD) and Implanol were categorized as long acting methods.

#### Demand for long acting contraceptives

It is determined by adding both the proportion of women who were using long acting contraceptive methods (met need) and proportion of women who were using any other short acting contraceptive methods but want to use long acting contraceptive methods (unmet need).

#### Unmet need for long acting contraceptive methods

Women who had the desired to use Implanol or IUCD for delay or limit pregnancy but did not use the methods due to any reason.

#### Knowledge of long acting contraceptive methods

If a woman mentioned one of the long acting contraceptive methods she was taken as knowledgeable.

#### Data collection procedures

Data was collected via face to face interview using structured questionnaire. Five data collectors and one supervisor were assigned during filed survey. Each data collector was assigned to each selected family planning delivery service unit. Exit interview was conducted after participants took contraceptive methods at the service delivery units.

### Data quality control measures

The data collection tool was first prepared in English and translated to the local language, Amharic and back translated to English by language expert to cheek for consistency. Pre-test was done on 5% of the respondents prior to the actual data collection period in another health center, which was not selected for the study and revision was done as necessary. Training was given for both data collectors and the supervisor on the data collection tools and techniques of interviewing. Daily supervision was done by the supervisor and principal investigator.

### Data processing and analysis

Data was cleaned for completeness and consistencies, coded and entered in to Epi info version 3.5.3 and transported to SPSS version 20 for analysis. The results were organized, summarized and presented using appropriate descriptive measures such as text, tables, graphs, frequencies and percentage. Associations between the outcome and independent variables were assessed by using odds ratio with 95% confidence interval. Bi-variable logistic regression was used to screen variables that had significant association with the outcome variable with P-value ≥ 0.2. These variables were entered in to multivariable logistic regression to assess the independent predictor of demand for long acting family planning methods. Variables which were significant at p-value 0.05 level and 95% CI were considered to be the determinant factors of demand for long acting family planning methods.

### Ethical considerations

Ethical clearance was obtained from Institutional Review Board of University of Gondar, College of Medicine and Health Sciences. Permission letter was obtained from Amhara National Regional State Health Bureau and all selected study health facilities. Informed verbal consent was obtained from each study participant after explaining the aim of the research. Individual participant records were coded on each respective questionnaire and accessed only by research team members. Confidentiality was maintained at all levels of the study.

## Results

### Socio-demographic characteristics of the respondents

A total of 487 women of aged 15-49 years participated in the survey with a response rate of 100%. Out of these, 421 (86.4%) were clients visited the health facilities for more than two times and the remaining 66 (13.6%) were new clients. One hundred eighteen (24.2%) of the participants were in the age group of 25-29 years. The mean age of the study participants was 29 years with SD ± 8 years. Four hundred thirty three (88.9%) of the respondents were currently married. The majority 444 (91.2%) of the respondents were Orthodox Christians. One hundred seventy seven (36.3%) of the participants were house wives followed by merchant 118 (24.2%). Regarding educational status, 139 (28.5%) of respondents had completed elementary school followed by college and above 117 (24%) [Table 1].

**Table 1:** **Socio-demographic characteristics of study participants in Debre-Tabor town, Northwest, Ethiopia, 2013, (n = 487)**

**Variable**	**Possible response**	**Frequency**	**Percentage**
Respondents age	15-19	38	7.8
	20-24	110	22.6
	25-29	118	24.2
	30-34	91	18.7
	35-39	66	13.6
	40-44	41	8.4
	45-49	23	4.7
Marital status	Single	54	11.1
	Married	433	88.9
Religion	Orthodox	444	91.2
	Others+	43	8.8
Occupation	House wife	177	36.3
	Governmental employ	83	17.0
	Daily Labour	20	4.1
	Student	89	18.3
	Merchant	118	24.2
Educational status	Unable to read and write	76	15.6
	Able to read and write	45	9.2
	Elementary school	139	28.5
	Secondary school	110	22.6
	Collage and above	117	25.1
Husband educational status	Unable to read and write	45	9.2
	Able to read and write	37	7.6
	Elementary school	101	20.7
	Secondary school	146	30.0
	Collage and Above	158	32.4
Ethnicity	Amhara	479	98.4
	Others*	8	1.6

+Others – Muslim, Protestant.

*Others -Tigrie and Oromo.

### Reproductive history of participants

One hundred ninety one (39.2%) of the respondents reported their first sexual debut was before 18 years while the remaining 296 (60.8%) was at the age of 18 years and above. The median age of the first sexual debut was 20 years and the mean age was 19 years with SD ± of 2.8 years. Currently one hundred ninety seven (40.2%) of the participants had no child, two hundred thirty five (48.5%) participants had one to four alive children and fifty five (11.3%) had five and more alive children. Three hundred thirty five (68.8%) of the participants had the intention to have a child in the future while 152 (31.2%) had no any intention to have a child in the future. About eighty six percent of the respondents ever heard about long acting family planning methods. Major source of information about long acting contraceptive methods was from health care providers accounting 354 (72.7%). Four hundred nineteen (86%) of the respondents were able to identify the source of long acting contraceptives methods. Of the total participants of the survey about 377 (77.4%) had general knowledge about long acting contraceptive methods on which only 187 (37.6%) had knowledge about Implanol and 137 (28%) had knowledge about IUCD [Table 2].

**Table 2:** **Frequency distribution of study participants by reproductive variables, in Debre-Tabor town, Northwest, Ethiopia, 2013**

**Variable**	**Response**	**Frequency**	**Percentage**
First sexual debut (487)	≤17	191	39.2
	≥18	296	60.8
Previous birth history (487)	Yes	290	59.5
	No	197	40.5
Status of previous birth (290)	Wanted	272	93.8
	Unwanted	18	6.2
Number of children (487)	0	197	40.2
	1-4	235	48.5
	≥5	55	11.3
Future birth intension (487)	Yes	335	68.8
	No	152	31.2
Ever heard about LACMs	Yes	420	86.2
	No	67	13.8
Ever heard one of the method	Implanol	384	78.9
	IUCD	299	53.2
Source of information about LACM	Radio	189	38.8
	Television	267	54.8
	Care providers	354	75.7
	News papers	44	9
	Neighbours	153	31.4
	Pamphlet	33	6.8
Identify source of information	Yes	419	86
	No	68	14
Source of methods	Hospital	348	71.5
	Higher clinic	134	27.5
	Health centre	354	72.7
	Small clinics	126	25.9
	Others*	61	12.5
Identify methods	Implanol	187	37.6
	IUCD	137	28.1

*Others –Drug vendors.

### Utilization of modern contraceptive methods

**Figure 1:**
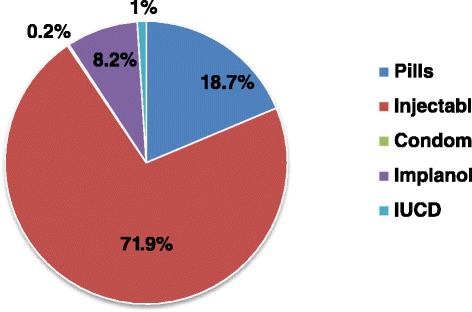
**Graphic presentation of participants by type of modern contraceptive users’ in Debre-Tabor town, Northwest Ethiopia, 2013.**

The current utilization rate of long acting contraceptive method in the town was 45 (9.2%). Of these, 40 (8.2%) were using Implanol and 5 (1%) intra uterine contraceptive device (IUCD). In the study area, the most widely used family planning methods were short acting contraceptives such as Injectables 350 (71.9%) and pills 91 (18.7%) [Figure 1].

**Figure 2:**
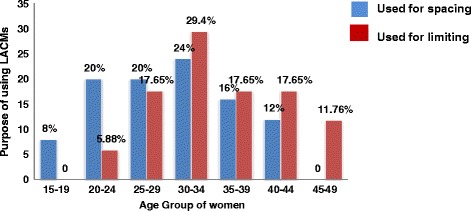
**Graphic presentation of current long acting contraceptive users by age group and purpose of utilization in Debre-Tabor town, Northwest Ethiopia, 2013.**

Utilization of long acting contraceptive methods varied with the age of respondents. The high proportions of users were observed in the age group of 30-34 years and lower in the extreme age groups [Figure 2].

### Unmet need for long acting contraceptive methods

**Figure 3:**
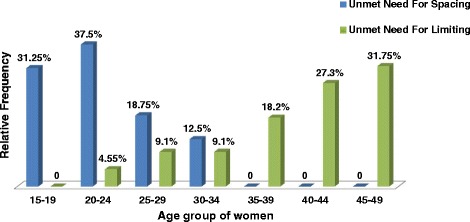
**Graphic presentation of unmet need for spacing and limiting for long acting contraceptive methods by age group in Debre-Tabor town, Northwest Ethiopia, 2013.**

Total unmet need for long acting contraceptive methods was found to be 7.8% (4.5% for spacing and 3.3% for limiting). Among thirty eight women having unmet need 22 (57.9%) wanted to delay pregnancy while 16 (42.1%) wanted to limit their number of children. Unmet need for long acting contraceptive methods was higher in the age group of 20-24 years and 45-49 years of mothers [Figure 3].

### Demand for long acting contraceptive methods

Demand for long acting contraceptive methods in the study area was 17%. This was the sum of current use of long acting contraceptive methods (met need) and the method desired but not used due to any reason (unmet need). Current long acting family planning users (met need) were 45 (9.2%) and unmet need were 38 (7.8%).

Out of the total short acting contraceptive method users, 39 (7.8%) of the participants were not preferring to use short acting family planning methods. Among these, 34 (89.5%) of the respondent had intensions to use implant and 4 (10.5%) of the respondents had intensions to use IUCD. The reasons for not using the preferred contraceptive methods were; fear of complications, husband/partner opposition, proven health problem and inconveniency of the methods [Table 3].

**Table 3:** **Distribution of current short acting contraceptive use and the reason for not preferring long acting contraceptive methods in Debre-Tabor town, Northwest Ethiopia, 2013**

**Variable**	**Response**	**Frequency**	**Percentage**
Currently used short acting contraceptive methods (442)	Preferred	404	91.4
	Not preferred	38	8.6
Reasons for not using implant (34)	Fear of complication	15	44.1
	Husband/Parent opposed	8	23.5
	Proven health problem	11	32.4
Reasons for not using IUCD (4)	Proven medical problem	2	50
	Husband/Partner opposition	1	25
	Inconveniency	1	25

### Service quality and inter-spousal communication

The results showed that during provision of the services, 459 (94.3%) clients were treated with respect, adequate privacy and dignity by the care providers. Almost all, 99.4%, of the respondents believed that the providers will keep their information confidential. Among clients who had received contraceptive methods, adequate information was given on how to choose among the methods, well explained side effects and got information what to do in case of problems for 467 (95.9%), 367 (75.4%) and 393 (80.7%) clients, respectively. More than half 264 (54.2%) of the participants treated very well by the health care providers while 223 (45.8%) were treated poorly Three hundred sixty four (74.7%) of the respondents had discussions about long acting contraceptive methods once or twice in the last few months and 123 (25.35%) of them had discussed more often. Three hundred sixty two (74.3%) of the participants responded that their husbands or partners approved the use of long acting contraceptive methods [Table 4].

**Table 4:** **Distribution of women by service quality, inter-spousal relations and cost of the services in Debre-Tabor town, Northwest Ethiopia, 2013**

**Variable**	**Response**	**Frequency**	**Percentage**
Client treated with dignity and respect	Yes	459	94.3
	No	28	5.7
Privacy kept during examination	Yes	484	99.4
	No	3	0.6
Believe information is kept confidential	Yes	484	99.4
	No	3	0.6
Previously discussed about long acting contraceptive methods	Yes	276	56.9
	No	211	43.1
Discussion with	Husband/Lovely	231	83.7
	Father/Mother	8	2.9
	Friends/ Neighbours	37	13.4
Who is decision maker to use the methods?	Self	187	38.4
	Husband	57	12.1
	Both together	241	49.5
Discussion with husband	More than two times	123	25.3
	Once/Twice	364	74.7
Husband attitude	Approve	362	74.3
	Opposed	72	14.8
	Don’t know	53	10.9
Convenience of the room	Convenient	414	85
	Inconvenient	73	15

### Factors associated with demand for long acting contraceptive methods

During bivariate logistic regression analysis; women occupational status, number of children, future birth intensions, ever heard about the methods, being treated with respect and dignity by care providers, myths and misconceptions on the methods, and frequency of discussions with husbands were found to be positively associated with demand for long acting contraceptive methods (P-value <0.05). These variables were included in multivariate analysis considering the cut-off point as P-value <0.2.

In the multivariable analysis, occupational status, future birth intensions, number of children, frequency of discussion with husband, being treated with respect and dignity and main decision maker to use were factors significantly associated with demand for long acting contraceptive methods. But, ever heard about the methods, myths and misconception about the methods, and had information from health centres were found to be negatively associated with demand for long acting contraceptive methods.

Occupational status was important predictor of demand for long acting contraceptive methods. Daily labourers had 3.87 times higher demand than house wives (AOR = 3.87, 95% CI = [1.06, 14.20]). Students had 2.64 times higher demand than house wives (AOR = 2.64, 95% CI = [1.27, 5.47]). Those mothers having future birth intensions had higher 2.17 times higher demand than those respondents who did not have future birth intensions (AOR = 2.17, 95% CI = [1.12, 4.23]). Women who have five or more children had 1.67 times higher demand for long acting contraceptive methods compared to women did not have children (AOR = 1.67, 95% CI = [1.58, 4.83]). Frequency of discussion with husbands on long acting contraceptive methods was also found to be significant predictors of demand for long acting contraceptive methods. Respondents who have more frequent discussions with their husbands or partners had 3.89 times higher demand than those who have discussion once or twice about long acting contraceptive methods (AOR = 3.89, 95% CI = [1.98, 7.65]). Respondents who were treated poorly by care providers had 0.42 times less demand for long acting contraceptive methods than those treated with respect and dignity (AOR = 0.42, 95% CI = [0.24, 0.74]). The other important predictor was main decision maker for using the methods, respondents who decided together with their husband had 2.73 times higher demand than those who decide by alone (AOR = 2.73, 95% CI = [1.40, 5.32]) [Table 5].

**Table 5:** **Multivariate analysis of demand for long acting contraceptive methods and determinant factors in Debre-Tabor town, Northwest Ethiopia, 2013**

**Variable**	**Demand for LACM**		**Crude OR**	**Adjusted OR**
	**Yes (%)**	**No (%)**	**(95% CI)**	**(95% CI)**
Occupational status				
House wife	27(15.3)	150(84.7)	1.00	1.00
Government employ	12(14.5)	71(85.5)	0.94(0.45, 1.96)	1.65(0.69, 3.92)
Daily Labours	6(30.0)	14(70.0)	2.38(0.84, 6.74)	3.87(1.06,14.20)***
Student	23(25.8)	66(74.2)	1.94(1.03, 3.62)	2.64(1.27, 5.47)***
Merchant	15(12.7)	103(87.3)	0.81(0.41, 1.60)	1.16(0.44, 3.06)
Future Birth intension				
Yes	48(14.3)	287(85.7)	1.00	1.00
No	35(33.0)	117(77.0)	1.79(1.10, 2.91)	2.17(1.11, 4.23)***
Number of children				
0	31(15.8)	165(84.2)	1.00	1.00
1-4	35(14.8)	201(85.2)	0.93(0.55, 1.57)	0.63(0.29, 1.38)
≥5	17(30.9)	38(69.1)	2.38(1.20, 4.74)	1.67(1.58, 4.83)***
Ever heard about the methods by radio				
Yes	21(11.1)	168(88.9)	2.10(1.23, 3.58)	
No	62(21.8)	236(79.2)	1.00	
Treatment status at health centres				
Very well	57(21.6)	207(79.4)	1.00	1.00
Poorly	26(11.2)	197(88.8)	0.48(0.29, 0.79)	0.42(0.24, 0.74)***
Number of discussion with husband				
More often	31(25.2)	92(74.8)	2.02(1.22, 3.34)	3.89(1.98, 7.65)***
Once/twice	312(85.7)	52(14.3)	1.00	1.00
Myths and misconceptions that Implant will cause anaemia				
Yes	18(40.9)	26(59.1)	1.00	
No	65(14.7)	378(85.3)	0.25(0.13, 0.48)	
Had information about the methods from health centres				
Yes	50(14.1)	304(85.9)	1.00	1.0
No	33(24.8)	100(75.2)	2.01(1.22, 3.29)	1.67(0.97, 2.89)
Myths and misconception that IUCD will cause permanent sterility				
Yes	11(30.6)	25(69.4)	1.00	
No	72(16.0)	379(84.0)	0.42(0.22, 0.80)	
Who is the decision maker to use?				
Self	30(16.1)	157(83.9)	1.00	1.00
Husband	5(8.5)	54(91.5)	0.49(0.18, 1.31)	0.62(0.21, 1.82)
Both together	48(19.8)	195(79.2)	1.30(0.79, 2.15)	2.73(1.40, 5.32)***

***_ Indicate variable did have significant association with demand P-Value <0.05.

## Discussion

This institutional based cross sectional study was attempted to assess the demand for long acting contraceptives and associated factors among family planning service users in Debre-Tabor town. The study showed that demand for long contraceptives was 17%. It is almost comparable with study done in Goba town and lower than a study conducted in Batu town Oromia region 18.2% and 24.4%, respectively [9,12]. But, have significant difference from study done in Iran which was 71% [1]. This high discrepancy might be due to the study setting, sample size and awareness of the participants on the methods.

In this study the utilization of long acting contraceptive methods was 9.2% which was in line with community based cross sectional study done in Goba town which was 8.04% [12]. But higher as compared with EDHS 2011 report [3]. The possible explanations might be due to difference in the study populations since EDHS used study populations including the rural area where they have low access to information and services.

Though long acting contraceptive methods which have high effectiveness, low burden to the users, good to avoid frequent visit for resupply, cost effective, and allowed rapid return to fertility after their removal, in this study there was under utilization of the methods which accounts implant 40(8.2%) and IUCD 5(1%). As study done in Tehuleder district of South Wollo zone showed implant (39%) and IUCD (8.7%) [13]. This discrepancy might be due to better awareness and promotion on the methods.

Currently, as the findings indicated higher proportions of participants were using implants than IUCD. This is in line with study done in Goba, Batu, Mekelle, Tehuleder districts of Ethiopia and EDHS 2011 report [3,11-13]. In this study large proportion of women were using short acting contraceptive methods for permanent limiting and spacing the number of children. This result was inconsistent with a study done Tehuledere district of south Wollo zone [14]. This might be due to the study set up and the design used in both studies.

The unmet need for long acting contraceptive methods in this study was 7.8%. It is almost similar with the study done in Goba town (9.4%). The reason for unmet need indicated by women who were not using long acting contraceptive methods but have a desire to use the methods were fear of complications, husband or partner opposition, proven other medical problems, and preference of short acting contraceptive methods.

According to this study, 86.2% of the respondent heard about long acting contraceptive methods of which 78.9% heard about implant and 53.2% about IUCD. It is similar with the study conducted in Goba town (86.6%) had information about long acting contraceptive methods [12]. This was higher compared to the EDHS 2011 report [3]. The possible difference might be that EDHS was a national study done both rural and urban populations and developing regions of the country.

Main source of obtaining these methods in the town were public health facilities and this was in line with EDHS 2011 report, study done in Mekelle, Batu and Goba towns [11-13].

Result of multivariate logistic regression analysis showed that occupational status, future birth intensions, number of children, being treated with respect and dignity by health care providers, frequency of discussions with husband or partner and the main decision maker to use these methods have significant association with demand for long acting contraceptive methods.

Those participants whose occupation was daily labour had 3.87 times higher demand for long acting contraceptive methods than house wives (AOR = 3.87, 95% CI = [1.06, 14.20]) which was similar with study done in Batu town [11]. Student family planning users had 2.64 times higher demand for long acting contraceptive methods than house wives (AOR = 2.64, 95% CI = [1.27, 5.47]). This finding was different from the study done in Batu [11] where student family planning users had 0.11 times demand for long acting contraceptive methods than house wives. The difference might be due to the time gap between the two studies i.e. students in this study might have better awareness and attitude to use these methods than students in the Batu town study four years ago.

Women who had more frequent discussions with their husband or partner about long acting contraceptive methods were had 3.89 times higher demand for the methods than those who had discussion once or twice (AOR = 3.89, 95% CI = [1.98, 7.65]). It was supported by the findings of the research done in Rwanda [15] which indicates that women who were discussing with their husband or partner about long acting contraceptive methods once or twice had 0.76 times less demand than women who discussed more often about the methods.

Women having five or more children had 1.67 times higher demand for long acting contraceptive methods compared to women who do not have children at all (AOR = 1.67, 95% CI = [1.58, 4.83]). It was similar with the finding of research done in Batu town [11] which indicated that women who had five or more children had 6 times higher demand for long acting contraceptive methods compared to women who didn’t have children at all (AOR = 5.85, 95% CI = [2.68, 12.8]).

Participants with no desire for more children in the future had 2.17 times higher demand for long acting contraceptive methods than those who wanted one or more children in the future (AOR = 2.17, 95% CI = [1.11, 4.23]). This was similar with the study done in Iran [1] in which women with no desire for future children had higher demand for long acting contraceptive methods compared to women having future childbearing intensions.

Women who decided together with their husband or partners had 2.73 times higher demand for long acting contraceptive methods than those who decided alone (AOR = 2.73, 95% CI = [1.40, 5.32]). This finding was almost in line with the study done in Butajira [16] in which women who had discussed and decided with their husband or partners were 2.2 times more likely to use the methods compared to those who decided alone [AOR 2.2 (95% CI: 1.8, 2.7)].

Clients treated with respect and dignity by care providers, had 42% higher demand for long acting contraceptive methods than those who were treated poorly (AOR = 0.42, 95% CI = [0.24, 0.74]) and this was similar with a study done in Kenya [17] which indicated that the health care provider quality towards to the client tends to increase the demand for long acting family planning methods.

### Limitation of the study

1. Since the study was institutional based and conducted only in the town among only family planning users, it might undermine generalizing the result to the majority general population including rural community and none users.
2. The study design is cross sectional; therefore it may be difficult to establish temporal relationship.
3. This study was conducted among only family planning service users in the government facilities; it may not representative to general population.

## Conclusions

Demand for long acting contraceptive methods among current family planning users was observed to be 17% in the town which was lower compared to the result of other studies. Even though there is adequate knowledge of long acting contraceptive methods among the study participant, there is a significant proportion of the mothers still having unmet need for the methods for both spacing and limiting. This shown that there is a risk of unwanted pregnancy and unsafe abortions.

The multivariate analysis revealed that variables such as occupational status, future birth intensions, number of alive children, clients treated well by the health care providers, number of discussion with husband about the methods and main decision maker for use had significant association with demand for long acting contraceptive methods.

### Recommendations

Concerned health managers and other support organizations should do more work to train health care providers to encourage women to make them able to discuss and persuade their partners on these methods.

Information education and communication (IEC) on long acting contraceptive methods should be provided to all and their partners’ women giving emphasis to those who have adequate number of children and women don’t have future birth intension.

Further comprehensive study is recommended to assess supply related factors, competency of service providers and considering private facilities, non users preferably community based study.
